# Association of gene polymorphism of SDF1(CXCR12) with susceptibility to HIV-1 infection and AIDS disease progression: A meta-analysis

**DOI:** 10.1371/journal.pone.0191930

**Published:** 2018-02-08

**Authors:** Jiwei Ding, Jianyuan Zhao, Jinming Zhou, Xiaoyu Li, Yanbin Wu, Mei Ge, Shan Cen

**Affiliations:** 1 Institute of Medicinal Biotechnology, Chinese Academy of Medical Sciences and Peking Union Medical School, Beijing, PR China; 2 School of Pharmacy, Shanghai Jiaotong University, Shanghai, PR China; University of Texas Rio Grande Valley, UNITED STATES

## Abstract

**Objectives:**

Genetic polymorphism of viral receptors is relevant to risks of HIV-1 infection, while it is still under debated whether the polymorphism of SDF1, a unique ligand for HIV-1 coreceptor CXCR4, is associated with HIV susceptibility and AIDS disease progression. Therefore, we provided an updated quantitative assessment by meta-analysis from 16 case-control and 7 cohort studies.

**Methods:**

Articles reporting the relationship between SDF1 polymorphism and HIV susceptibility or AIDS progression were retrieved from PubMed, Embase and Ovid electronic databases up to Apr 2017. Data were pooled by odds ratios (ORs) for HIV-1 infection with 95% confidence intervals (CIs) and summary relative hazards (RHs) for AIDS progression with 95% CIs using 1987 Center for Disease Control (CDC) case definition of AIDS (CDC87) and 1993 Center for Disease Control (CDC) case definition of AIDS (CDC93) and death as endpoints.

**Results:**

As a result, 16 studies regarding susceptibility to HIV-1 infection with 2803 HIV-infected patients and 3697 healthy individuals and 7 studies regarding disease progression with 4239 subjects were included in the meta-analysis. For risks of infection, no evidences indicated SDF1 polymorphism was associated with the risk of HIV-1 infection in all genetic models (recessive model: OR = 0.94, 95% Cl: 0.75–1.17; homozygous model: OR = 0.89, 95% Cl: 0.70–1.15; heterozygous model: OR = 1.06, 95% Cl: 0.83–1.35; allele model: OR = 0.95, 95% Cl: 0.79–1.13), Furthermore, we failed to find an delayed AIDS progression except in some specific cohorts including MACS cohorts (RH = 0.38, 95% Cl: 0.17–0.59 for time to AIDS; RH = 0.27, 95% Cl: 0.07–0.46 for time to death at the study entry).

**Conclusions:**

Overall, no significant association was found between SDF1 polymorphism and HIV susceptibility. A protective effect of SDF1 on AIDS progression and death was seen especially in two studies based on the same cohorts. In conclusion, SDF1 polymorphism exerts a moderate protective effect against AIDS disease deterioration in some specific populations.

## Introduction

Viral entry of human immunodeficiency viruses (HIV) required CD4 molecule and one member of CC or CXC chemokine-receptor families as co-receptors on cell membrane of lymphocytes. Genetic polymorphism of chemokines or chemokine-receptors was reported to be relevant to risks of human immunodeficiency viruses infection and disease progression. HIV viruses can be categorized to two major types according to the usage of co-receptors: R5 tropic viruses that use CC chemokine receptor (CCR) 5 and X4 tropic viruses that use CXC chemokine receptor (CXCR) 4. The stromal cell derived factor (SDF1), also designated as CXCR12, is the unique ligand for one of HIV-1 coreceptor CXCR4. A guanine to adenine mutation at nucleotide position 801 in the 3’ untranslated region (UTR) of SDF1 (abbreviated as SDF1-3’A) was reported to confer resistance to HIV infection [1, 2] and delay disease progression in homozygous individuals. The authors proposed that SDF1-3’A mutation resulted in higher production of plasma SDF1 level, thereby preventing X4-tropic HIV viruses from binding to CXCR4 receptor [3]. However, conflicting results were also reported by several other groups [4, 5]. For instance, in a cohort of 1090 individuals from the US Tri-Service HIV Natural History Study, SDF1-3’A homozygotes showed an accelerated disease progression using the CDC AIDS definition and more rapid death [6]. Otherwise, no significant association between SDF1-3’A and HIV infection [7–10] or AIDS progression was found in other researches. To disentangle the discrepancy, meta-analysis were conducted in our research. We evaluated the association of SDF1 polymorphism with HIV susceptibility and AIDS disease progression by 16 case-control studies and 7 cohort studies. It is the first time to evaluate the association of SDF1 polymorphism and AIDS progression based on different sources of research subjects.

## Methods

### Publication search

The meta-analysis followed the Preferred Reporting Items for Systematic Reviews and Meta-analysis criteria [11]. A comprehensive literature search was systematically conducted using the electronic database including PubMed, Embase or Ovid for all articles on the association of SDF1 polymorphism and HIV susceptibility or AIDS disease progression, covering literatures published until April 2017. The combination of the following key words was used: “SDF1 or CXCL12”, “polymorphism or variant or mutation” and “HIV infection or AIDS or human immunodeficiency virus”. For instance, the full search strategy in the Pubmed database is: text word = (("chemokine cxcl12"[MeSH Terms] OR“SDF1"[MeSH Terms]) AND (polymorphism[tiab] OR variant[tiab] OR mutation[tiab]) AND (HIV[tiab] OR human immunodeficiency virus[tiab] OR AIDS[tiab])). Furthermore, additional literature was collected manually from reference lists of relevant publications to make sure all potential eligible publications were retrieved. The search was restricted to language in English and performed by two independent researchers.

### Inclusion and exclusion criteria

Studies were included if they met the inclusion criteria as follows: (1) case-control or cohort study; (2) provide contingency table in which the number of HIV+/- infected patients with each genotype was present in the content of HIV susceptibility. To analyze AIDS disease progression, relative hazards (RHs) with 95% CIs was provided by means of Cox proportional hazards models; (3) fulfil the Hardy-Weinberg Equilibrium (HWE). In addition, the major exclusion criteria were as follows: (1) lack of enough controls; (2) reviews, conference abstracts or case report; (3) poor study design or quality of paper; (4) small sample study; (5) not human studies; (6) duplicates of previous literature.

### Data extraction and quality assessment

Two investigators reviewed and extracted information from all the eligible publications independently according to the exclusion and inclusion criteria. Disagreement was solved by a discussion with a third investigator to reach a consensus on all the extracted information. For each article, the following characteristics were collected from each study: author name, year of publication, country, ethnicity, genotyping method, number of case and controls in HIV-infected patients and healthy controls, respectively and RHs of disease progression or death with 95% CIs. When RHs were provided in a graph, we obtained exact values using GetData Graph Digitizer 2.26 (http://getdata-graph-digitizer.com).

The quality of studies was assessed by the validated Newcastle-Ottawa Scale (NOS) for nonrandomized studies. NOS allot eight points to each study (four for quality of selection, one for comparability, and three for outcome). A study can be allotted for a maximum of one star in quality of selection and outcome categories and a maximum of two stars in comparability category. We evaluated studies with scores of more than 6 as high-quality studies.

### Statistical analysis

Crude ORs with 95% CIs were used to assess the strength of association of SDF1 polymorphism and susceptibility of HIV infection, under four different genetic model (recessive model, allele model, homozygous model and heterozygous model), based on genotype frequency distribution in cases and controls. A OR value >1 indicated a significant increase in susceptibility to HIV-1 infection and OR value <1 stood for a protective effect of HIV infection. The goodness-of-fit chi-square test was adopted to check the HWE. Heterogeneity was assessed by χ^2^ based Q test. If P value of heterogeneity >0.05, indicated an absence of heterogeneity between studies, then we adopted a fixed-effect model (Mantel-Haenszel) to assess the pooled ORs. If P value of heterogeneity <0.05, indicated a presence of heterogeneity between studies, then we adopted a random-effect model (DerSimonian and Laird) to assess the pooled ORs. Summary RR estimates were calculated using random-effect model. Heterogeneity between studies was evaluated by Q test and I^2^ statstic. We predefined heterogeneity (I^2^< 25% for low, 25% < I^2^< 50% for moderate, and I^2^ > 50% for high). In order to explore the source of heterogeneity, a sensitivity analysis and subgroup analysis were conducted. These included subgroup analysis stratified based on sources of research subjects, ethnicity, seroconversion and whether or not the studies was adjusted for potential important confounders. Publication bias was tested by Egger’s test or Begg’s test and described by a funnel plot. Probable publication bias was corrected using “trim and fill” method. All statistical analysis except for the funnel plot was performed in Stata 14.0 software package. The funnel plot was conducted in Revman 5.3. A value less than 0.05 denoted a statistical significance.

## Results

### Study characteristics

**Fig 1:**
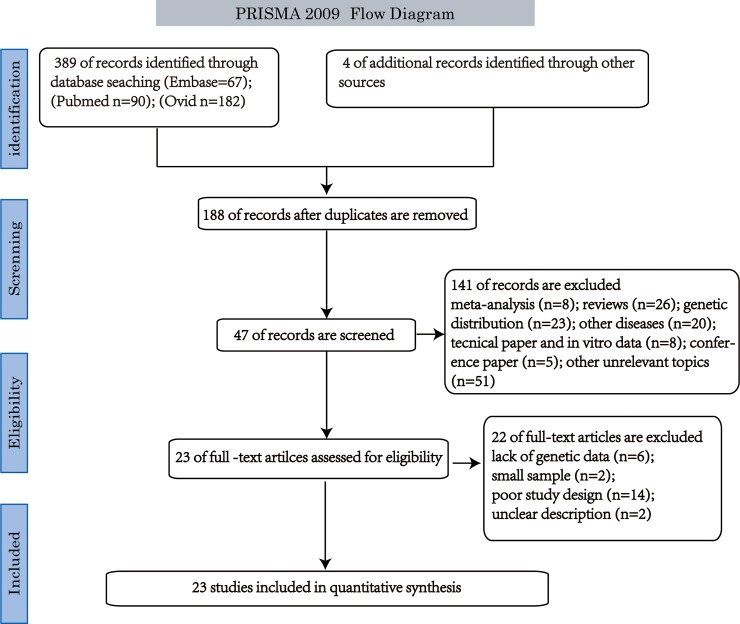
Selection process of studies included in meta-analysis.

As shown in Fig 1, a total of 393 publications were identified by an initial search from the electronic databases and other sources. After reviewing the title, abstract and the full paper, 23 studies were enrolled for meta-analysis. 16 studies were found to fulfill the eligibility criteria for the current meta-analysis with susceptibility to HIV infection including 2803 patients and 3697 healthy controls [2, 4, 5, 7, 8, 10, 12–22]. 7 studies were found to fulfill the eligibility criteria for the association analysis of SDF1 polymorphism and AIDS progression including 4239 HIV-infected patients [23–31]. The prevalence of SDF-1’3A allele differs among races, ethnic groups, and risk groups with its consequences in differential susceptibility to infection. The frequency of SDF-1’3A allele was 29.1% and 28.6% in HIV-1 infected patients and healthy controls. Besides, the distribution of genotypes was consistent for Hardy-Weinberg Equilibrium in all involved studies. Among them, there were seven studies for Asians, five studies for Americans, six studies for Europeans, three studies for Latin-Americans and two for Africans. All studies included healthy controls, while seven included HIV exposed but seronegative (HESN) controls. Only data of HESN controls was adopted in the analysis when both HESN controls and healthy controls were available, considering HESN controls might provide a better paired comparison with HIV infected patients than healthy controls.

There were 9 high quality case-control studies, as determined by Newcastle-Ottawa Scale score of 6 or higher (S1 Table) and 4 high quality cohort studies (S2 Table). Studies concerning the susceptibility of HIV infection were divided into two subgroups based on their NOS scores. All the studies relevant to AIDS disease progression were included into the meta-analysis regardless of NOS scores. The summary of characteristics of eventual included studies relevant to HIV susceptibility and AIDS progression was listed in the S3 and S4 Tables, respectively.

### Meta-analysis

**Fig 2:**
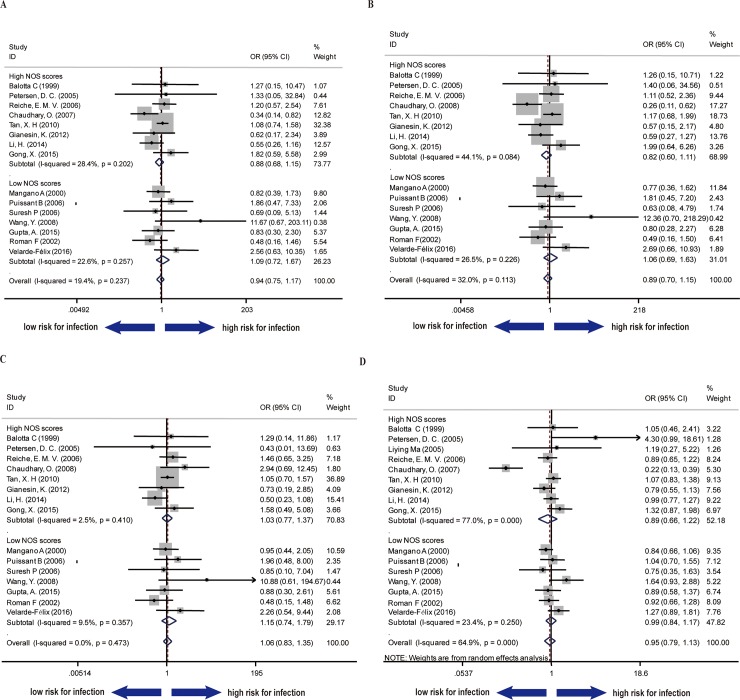
Forest plot of the association of SDF1-3’A and HIV susceptibility in different genetic models. (A)recessive model (B) homozygous model (C) heterozygous model (D) allele model. Studies were divided into two subgroups (High NOS scores group and Low NOS scores group) as indicated.

After pooling the data from 16 studies regarding susceptibility of HIV-1 infection, the results were calculated using four genetic models (recessive model, homozygous model, heterozygous model and allele model), respectively. The studies were divided into two subgroups based on their NOS scores, thereby a more convincing conclusion could be made using both high quality evidence and all data collected. Overall, our results did not show a significant association between SDF1 polymorphism and susceptibility to HIV infection in all four models (recessive model: pooled OR = 0.94, 95% Cl: 0.75–1.17, P = 0.237; homozygous model: pooled OR = 0.89, 95% Cl: 0.70–1.15. P = 0.113; heterozygous model: pooled OR = 1.06, 95% Cl: 0.83–1.35, P = 0.473; allele model: pooled OR = 0.95, 95% Cl: 0.79–1.13 P = 0.000) (Fig 2). The conclusion was consistent when only data from literatures of high NOS scores were pooled.

**Fig 3:**
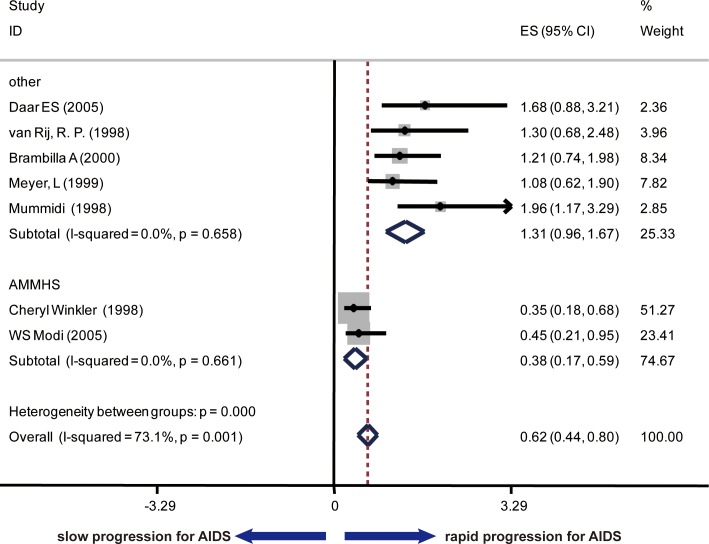
Forest plot of the association of SDF1-3’A and AIDS progression according to CDC87 criteria stratified by sources of research subjects.

**Fig 4:**
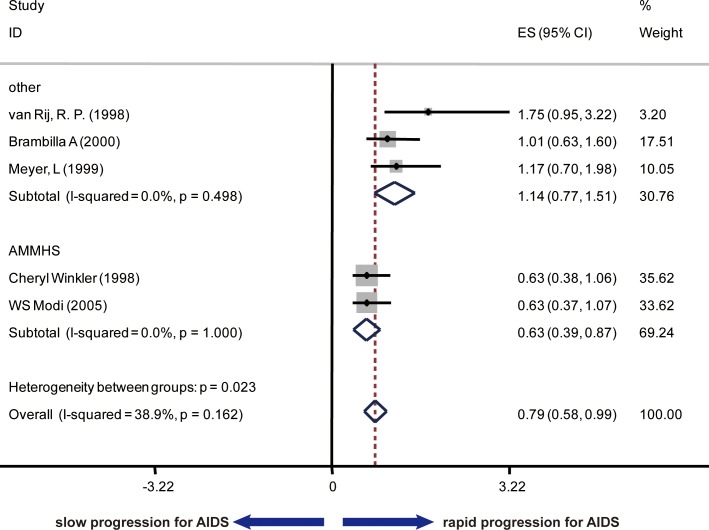
Forest plot of the association of SDF1-3’A and AIDS progression according to CDC93 criteria stratified by sources of research subjects.

**Fig 5:**
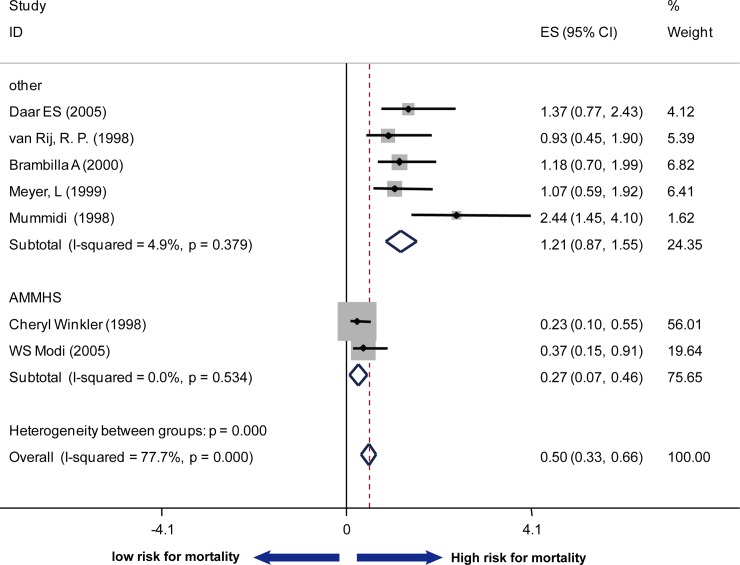
Forest plot of the association of SDF1-3’A and death stratified by sources of research subjects.

To analyze the association of SDF1 polymorphism and AIDS disease progression and survival, another 7 cohort studies were included and the data were extracted about the RHs of AIDS occurrence according to CDC87 and/or CDC93 and the RHs of death. The summary RHs was 0.62 (95% Cl: 0.44–0.80; I^2^ = 73.1%) with the data for AIDS according to CDC87 as the endpoint (Fig 3), 0.79 (95% Cl: 0.58–0.99; I^2^ = 38.9%) with the data for AIDS according to CDC93 as the endpoint (Fig 4) and 0.50 (95% Cl: 0.33–0.66; I^2^ = 77.7%) with the data for death (Fig 5). Considering the high heterogeneity, all studies were divided into two subgroups: one group included the studies conducted by Cheryl Winkler and WS Modi (Group 1), of which research subjects were selected from five epidemiologic cohorts (MACS, SFCC, MHCS, HGDS and ALIVE cohorts, we used AMMHS as an abbreviation), and in another group (Group 2), 5 studies conducted research based on multiple cohorts distinguish from the first group. Since each cohort belonged to different risk groups, race, access to medical care, route of exposure and HLA polymorphism, when both unadjusted and adjusted RHs were provided, only adjusted RHs were counted into the meta-analysis. In Group 1, the summary RH was 0.38 (95% Cl: 0.17–0.59; I^2^ = 0.0%, P = 0.661) with the data for AIDS (CDC87) as the endpoint, 0.63 (95% Cl: 0.39–0.87; I^2^ = 0.0%, P-1.000) with the data for AIDS (CDC93) as the endpoint and 0.27 (95% Cl: 0.07–0.46; I^2^ = 0.0%, P = 0.534) with the data for death as the endpoint. In contrast, no significant association was found between SDF1-3’A and AIDS clinical progression or death in Group 2. CCR5 and CCR2 polymorphism have been demonstrated to be a risk factor in disease progression, which underscored the importance of controlling for confounding factors (S4 Table). To make a more precise conclusion, RHs adjusted for CCR5 and CCR2 tropism was also summarized. In agreement with the previous conclusion, no significant association of SDF1-3’ A polymorphism with disease progression was found except in AHMMS cohort (S1 Fig).

### Publication bias

**Fig 6:**
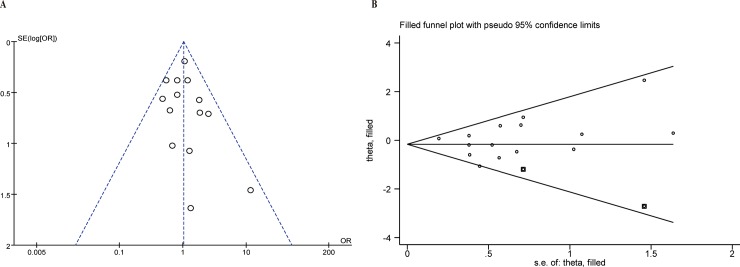
Funnel plot of publication bias test. Funnel plot without (A) or with (B) “trim and fill” for recessive model.

Egger’s weighted regression method and Begg’s rank correlation method was used to statistically assess the publication bias for the 16 studies involved in the susceptibility analysis. There was no evidence of publication bias (Egger’ test P = 0.461, Begg test P = 0.198, recessive model). Other genetic models was checked by Begg and Egger test’s in the same fashion, neither publication bias nor small sample effect was detected in other three models (allele model: Egger’ test P = 0.818, Begg test P = 0.392; homozygous model: Egger’ test P = 0.401, Begg test P = 0.235; heterozygous model: Egger’ test P = 0.371, Begg test P = 0.166). In addition, visual inspection of funnel plot asymmetry has been performed for recessive model by Revman5.3 (Fig 6A). However, the absence of points in the bottom left side indicated probable publication bias for small samples studies. To be more prudent, “trim and fill” method was conducted to reduce possible bias. The result revealed that two studies might be missing (Fig 6B) and pooled ORs were not changed significantly (OR = 0.88, 95% Cl: 0.70–1.11) after adjusted bias, which suggested our results were reliable. Based on these quantitive results of publication bias, we concluded that the slight publication bias did not affect our overall results.

## Discussion

A potential role of host genetic factors in predisposition to HIV infection has been described in several reports [32, 33]. SDF1, the unique ligand of HIV coreceptor CXCR4 was reported to be associated with host susceptibility and AIDS progression but the conclusions were controversial. In the present study, our meta-analysis on the association of SDF1-3’A and HIV infection risk involved 16 eligible studies with 2803 HIV-infected patients and 3697 healthy individuals. The strength of our study was based on the accumulation of published studies with high scores rated by Newcastle-Ottawa Scale. Two previous studies [34, 35] pertinent to the association of SDF1-3’A and HIV infection risk lacked the important quality control process of literatures and variety of genetic models. Moreover, we hypothesized that HIV exposed seronegative (HESN) individuals were more powerful controls than randomly selected healthy controls, thus we choosed HESN individuals as control if the data of HESN individuals were available. The studies demonstrated that SDF1-3’A has little protective effect against HIV infection when all subjects were included in the statistical process in all four genetic models (recessive model, homozygous model, heterozygous model and allele model).

Whether SDF1 polymorphism accelerates or delays AIDS disease progression is a matter of ongoing debate. Data of RHs and its 95% CIs for disease progression (according to CDC87 and CDC93 criteria for AIDS separately) and death was obtained from original paper. Pooled RHs were calculated using random-effect model weighted by inverse variance method. We included 7 studies with 4239 HIV-infected patients. Considering the high statistical heterogeneity when all samples were counted into, we performed subgroup analysis for exploring the sources of heterogeneity. The heterogeneity was not reduced when the analysis were stratified by ethnicity, country, year of publication or study design (I^2^>50, p<0.01). Of note, two studies which demonstrated the SDF1-3’A polymorphism had a protective effect against AIDS progression based their researches on the same cohorts. So we divided the studies into two groups: in the first group, the subjects were selected from five epidemiologic cohorts (AMHHS cohorts), and in another group, the subjects were selected from other cohorts distinct from the first group. In AMHHS cohort, the RHs for disease progression (CDC87) and death is 0.38 (95% Cl: 0.17–0.59; I^2^ = 0.0%) and 0.27 (95% Cl: 0.07–0.46; I^2^ = 0.0%) respectively. In contrast, the RHs for both disease progression and death became not significant in another group. The positive effect of SDF1-3’A in AMHHS combined cohort is mainly attributable to that of MACS cohort. Of note, MACS cohort is a US-based ongoing prospective study of HIV-infected adult (ages 18–70) homosexual and bisexual men in Baltimore, Chicago, Pittsburgh and Los Angeles enrolled from 1984 to 1991. The racial distribution is 83.3% Europe American, 10% African American, 5% Hispanic and 2% others. A significant majority of these men in the cohort reported having 50 or more lifetime sexual partners, and over 80% had engaged in receptive anal intercourse with at least some of their partners in the previous two years [36]. In agreement with our conclusion, WS Modi reported that none of the end points was significantly associated with SDF1-3’A in the MHCS or any other individual cohort. We observed different effects of genetic risk for AIDS progression between MACS cohorts and other multiple cohorts. This is expected since AIDS disease progression is a complex process with various confounders involved (different routes of exposures, different ART therapy regimens and even different environmental factors). Besides, cohort may have its inherent ascertainment bias. For instance, seroprevalent studies underrepresented the rapid progressors and overrepresented the slow and nonprogressors. Taken together, we conclude that SDF1-3’A exerted a moderate influence on AIDS progression in specific populations.

Although meta-analysis is a powerful tool, inherent limitations of this study should be addressed. Firstly, only studies written in English are included which might bias conclusions in our study. Secondly, with regard to the association of SDF1-3’A and AIDS progression, only 7 reports are included and some of them lack a good control of confounders, limited number of studies might influence the eventual result. Finally, different exposure routes (bisexual or homosexual contact, intravenous drug use, etc) is a priority for infection risk and analysis of infection routes should be performed, however, the detailed data of HIV infected patients by different routes are lacking. Nevertheless, our study indicates SDF1 polymorphism exert little effect on HIV-1 infection whereas it exert a moderate influence AIDS progression in some specific populations.

Our study conforms to the PRISMA statement [37] and the meta-analysis-on-genetic-association studies-form, the checklists are provided in S5 and S6 Tables.

## Supporting information

S1 Fig Forest plot of adjusted RHs. (A) Forest plot of RHs of SDF1-3’A homozygosity for AIDS progression (CDC87) which are adjusted for coreceptor tropism and stratified by cohorts. (B) Forest plot of the RHs of SDF1-3’A homozygosity for death which are adjusted for coreceptor tropism and stratified by cohorts.
(TIF)

S1 Table Quality assessment of the included case-control studies by Newcastle-Ottawa Scale. (TIF)

S2 Table Quality assessment of the included cohort studies by Newcastle-Ottawa Scale. (TIF)

S3 Table The summary of characteristics of eventual included studies relevant to HIV susceptibility. (TIF)

S4 Table The summary of characteristics of eventual included studies relevant to AIDS progression. (TIF)

S5 Table PRISMA 2009 checklist. (DOC)

S6 Table Meta-analysis on genetic association studies form. (DOCX)
