# Supplementary figures and images for: Association of gene polymorphism of SDF1(CXCR12) with susceptibility to HIV-1 infection and AIDS disease progression: A meta-analysis
Source: PLoS One. 2018 Feb 8;13(2):e0191930. doi: 10.1371/journal.pone.0191930 (PMC5805253; doi:10.1371/journal.pone.0191930)

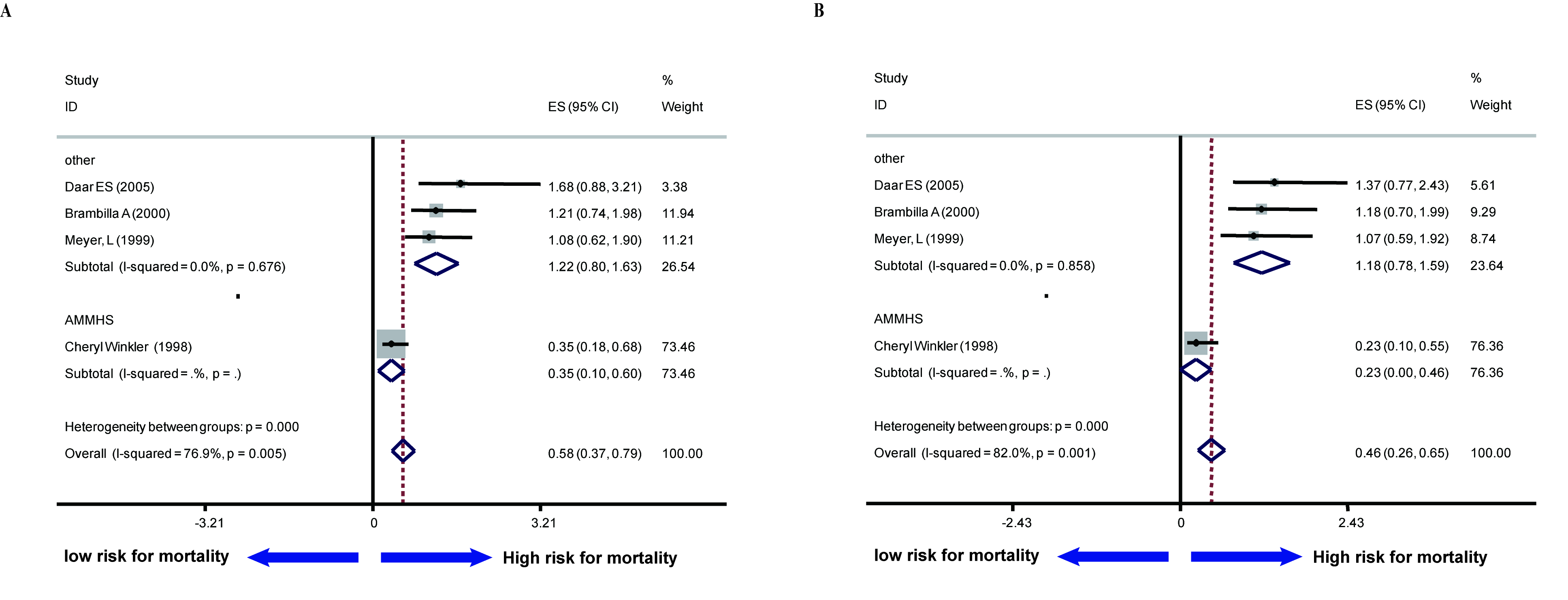

Supplement: S1 Fig — (A) Forest plot of RHs of SDF1-3’A homozygosity for AIDS progression (CDC87) which are adjusted for coreceptor tropism and stratified by cohorts. (B) Forest plot of the RHs of SDF1-3’A homozygosity for death which are adjusted for coreceptor tropism and stratified by cohorts. (TIF) [file pone.0191930.s001.tif]

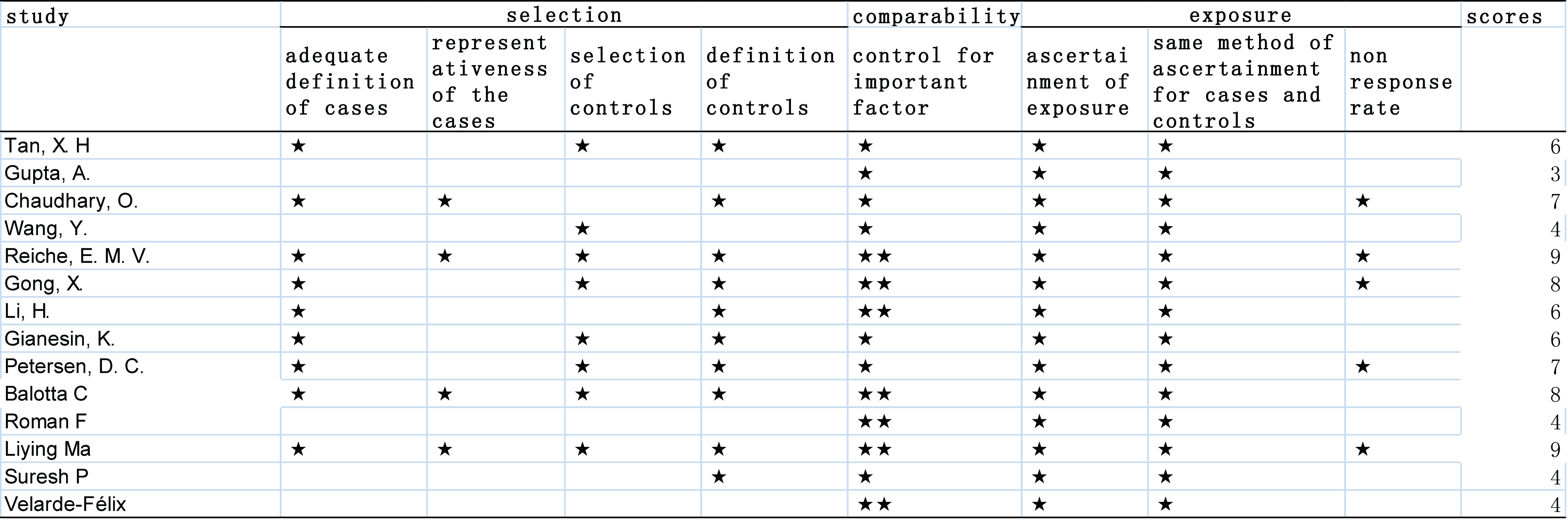

Supplement: S1 Table — (TIF) [file pone.0191930.s002.tif]

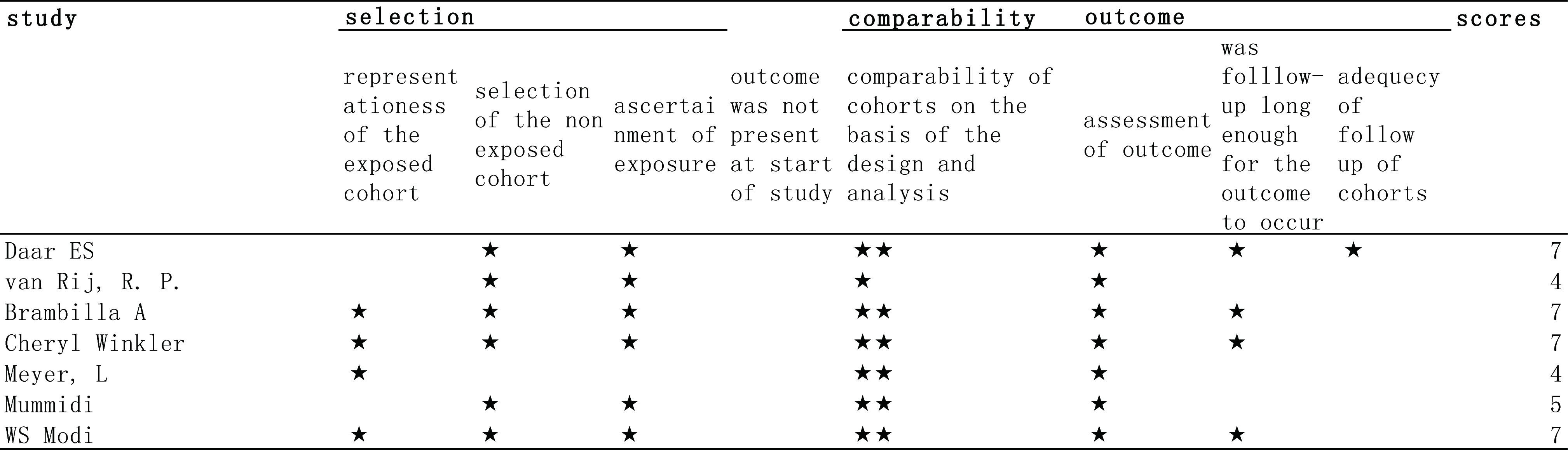

Supplement: S2 Table — (TIF) [file pone.0191930.s003.tif]

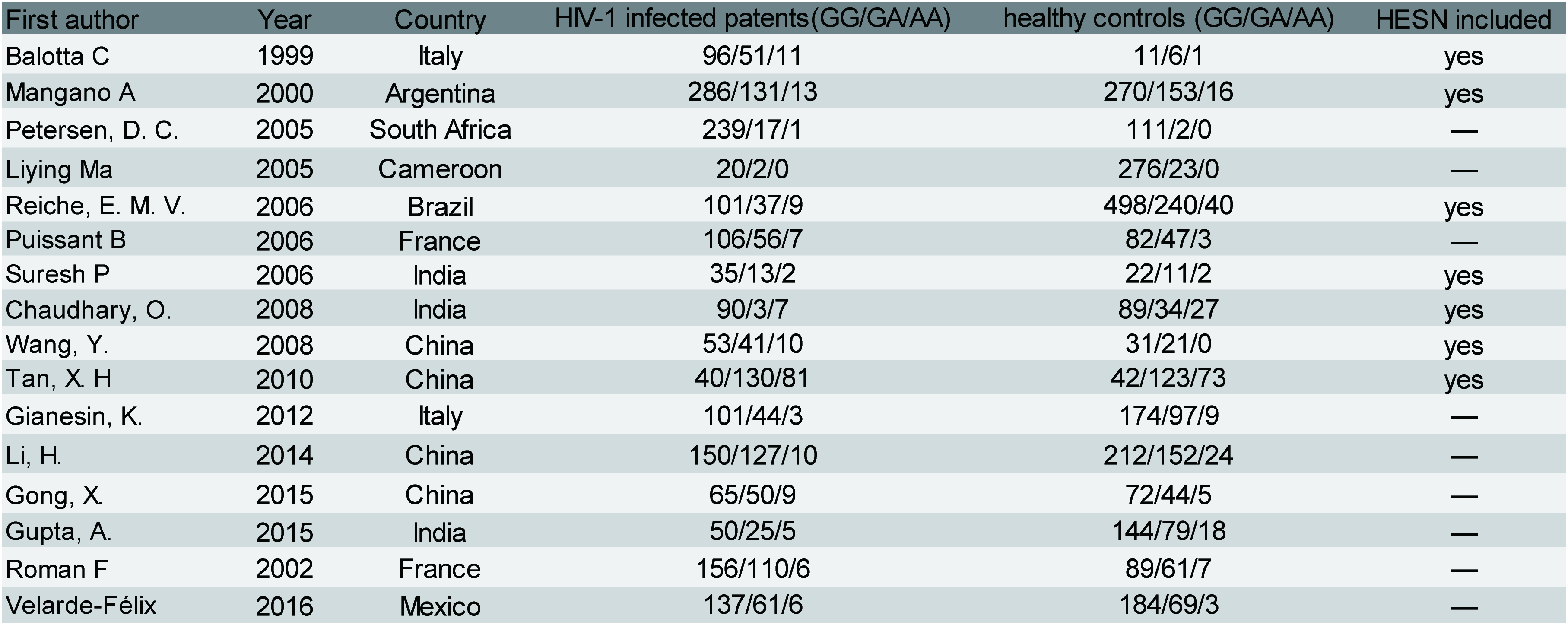

Supplement: S3 Table — (TIF) [file pone.0191930.s004.tif]

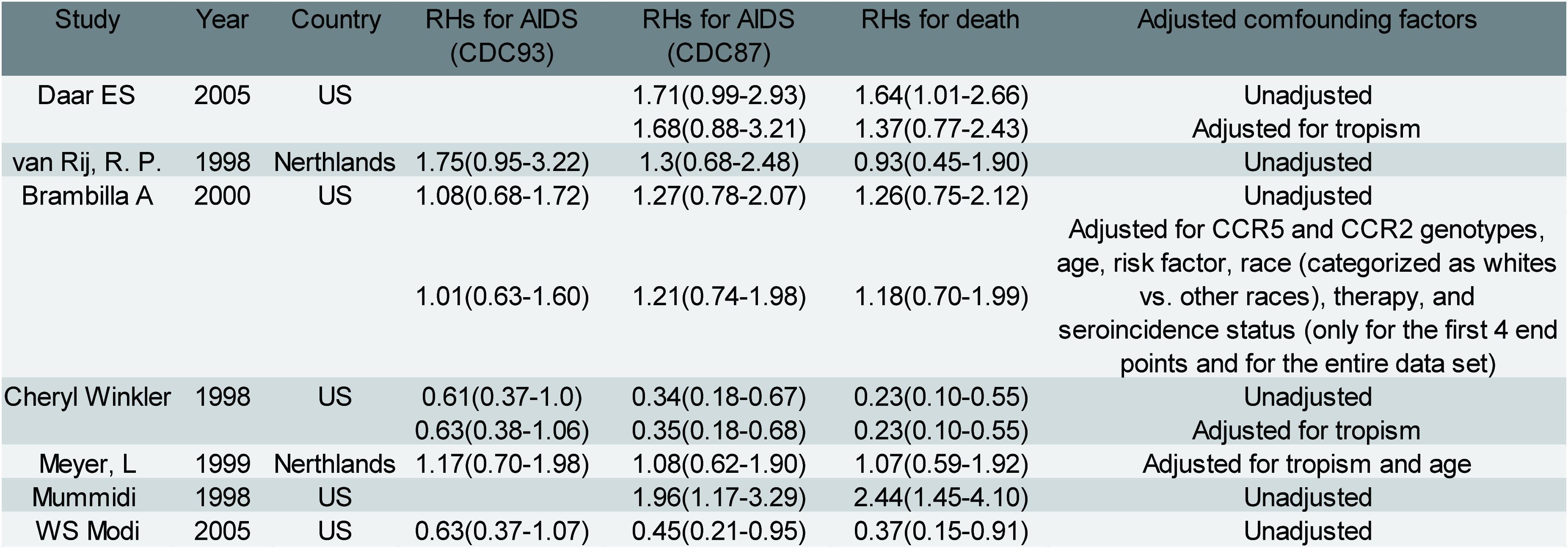

Supplement: S4 Table — (TIF) [file pone.0191930.s005.tif]
